# Assessing Speaking Proficiency: A Narrative Review of Speaking Assessment Research Within the Argument-Based Validation Framework

**DOI:** 10.3389/fpsyg.2020.00330

**Published:** 2020-02-27

**Authors:** Jason Fan, Xun Yan

**Affiliations:** ^1^Language Testing Research Centre, The University of Melbourne, Melbourne, VIC, Australia; ^2^Department of Linguistics, University of Illinois at Urbana-Champaign, Champaign, IL, United States

**Keywords:** speaking assessment, speaking proficiency, argument-based validation framework, research methods, narrative review

## Abstract

This paper provides a narrative review of empirical research on the assessment of speaking proficiency published in selected journals in the field of language assessment. A total of 104 published articles on speaking assessment were collected and systematically analyzed within an argument-based validation framework (Chapelle et al., 2008). We examined how the published research is represented in the six inferences of this framework, the topics that were covered by each article, and the research methods that were employed in collecting the backings to support the assumptions underlying each inference. Our analysis results revealed that: (a) most of the collected articles could be categorized into the three inferences of *evaluation, generalization*, and *explanation*; (b) the topics most frequently explored by speaking assessment researchers included the constructs of speaking ability, rater effects, and factors that affect spoken performance, among others; (c) quantitative methods were more frequently employed to interrogate the inferences of *evaluation* and *generalization* whereas qualitative methods were more frequently utilized to investigate the *explanation* inference. The paper concludes with a discussion of the implications of this study in relation to gaining a more nuanced understanding of task- or domain-specific speaking abilities, understanding speaking assessment in classroom contexts, and strengthening the interfaces between speaking assessment, and teaching and learning practices.

## Introduction

Speaking is a crucial language skill which we use every day to communicate with others, to express our views, and to project our identity. In today's globalized world, speaking skills are recognized as essential for international mobility, entrance to higher education, and employment (Fulcher, 2015a; Isaacs, 2016), and are now a major component in most international and local language examinations, due at least in part to the rise of the communicative movement in language teaching and assessment (Fulcher, 2000). However, despite its primacy in language pedagogy and assessment, speaking has been considered as an intangible construct which is challenging to conceptualize and assess in a reliable and valid manner. This could be attributable to the dynamic and context-embedded nature of speaking, but may be also due to the various forms that it can assume (e.g., monolog, paired conversation, group discussion) and the different conditions under which speaking happens (e.g., planned or spontaneous) (e.g., Luoma, 2004; Carter and McCarthy, 2017). When assessing speaking proficiency, multiple factors come into play which potentially affect test takers' performance and subsequently their test scores, including task features, interlocutor characteristics, rater effects, and rating scale, among others (McNamara, 1996; Fulcher, 2015a). In the field of language assessment, considerable research attention and efforts have been dedicated to researching speaking assessment. This is evidenced by the increasing number of research papers with a focus on speaking assessment that have been published in the leading journals in the field.

This prolonged growth in speaking assessment research warrants a systematic review of major findings that can help subsequent researchers and practitioners to navigate the plethora of published research, or provide them with sound recommendations for future explorations in the speaking assessment domain. Several review or position papers are currently available on speaking assessment, either reviewing the developments in speaking assessment more broadly (e.g., Ginther, 2013; O'Sullivan, 2014; Isaacs, 2016) or examining a specific topic in speaking assessment, such as pronunciation (Isaacs, 2014), rating spoken performance (Winke, 2012) and interactional competence (Galaczi and Taylor, 2018). Needless to say, these papers are valuable in surveying related developments in speaking proficiency assessment and sketching a broad picture of speaking assessment for researchers and practitioners in the field. Nonetheless, they typically adopt the traditional literature review approach, as opposed to the narrative review approach that was employed in this study. According to Norris and Ortega (2006, p. 5, cited in Ellis, 2015, p. 285), a narrative review aims to “scope out and tell a story about the empirical territory.” Compared with traditional literature review which tends to rely on a reviewer's subjective evaluation of the important or critical aspects of the existing knowledge on a topic, a narrative review is more objective and systematic in the sense the results are usually based on the coding analysis of the studies that are collected through applying some pre-specified criteria. Situated within the argument-based validation framework (Chapelle et al., 2008), this study is aimed at presenting a narrative review of empirical research on speaking assessment published in two leading journals in the field of language assessment, namely, *Language Testing* (LT) and *Language Assessment Quarterly* (LAQ). Through following the systematic research procedures of narrative review (e.g., Cooper et al., 2019), we survey the topics of speaking assessment that have been explored by researchers as well as the research methods that have been utilized with a view to providing recommendations for future speaking assessment research and practice.

## Theoretical Framework

**Figure 1:**
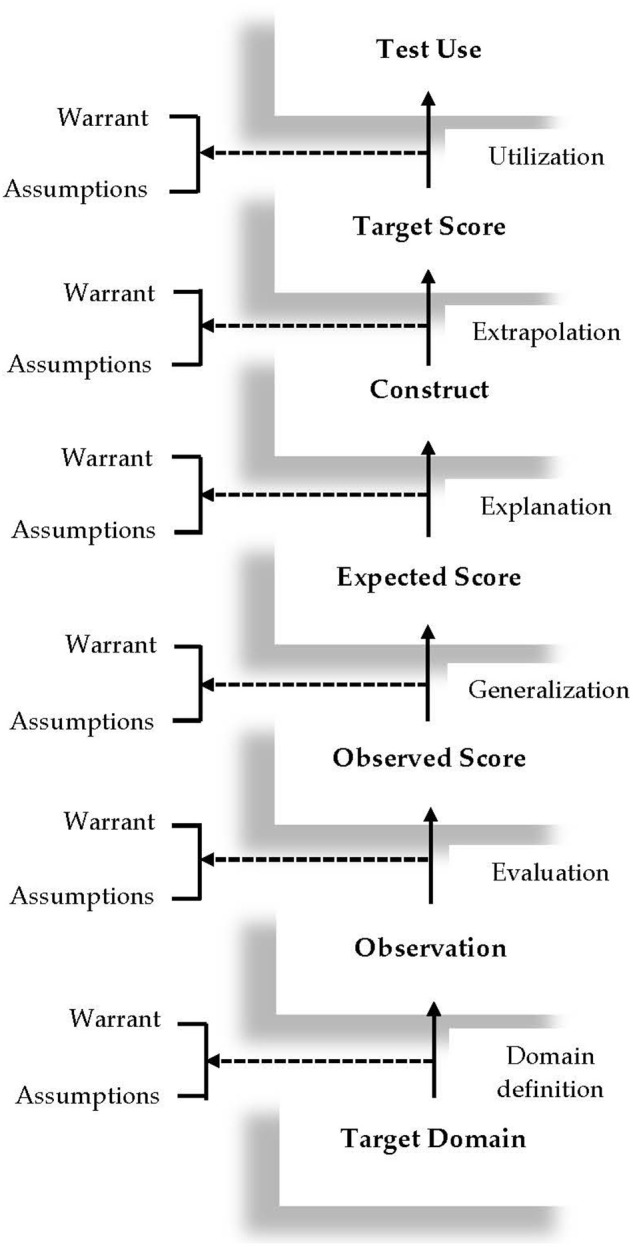
The argument-based validation framework (adapted from Chapelle et al., 2008, p. 18).

Emerging from the validation of the revised Test of English as a Foreign Language (TOEFL), the argument-based validation framework adopted in this study represents an expansion of Kane's (2006) argument-based validation model, which posits that a network of inferences needs to be verified to support test score interpretation and use. A graphic display of this framework is presented in Figure 1. As shown in this figure, the plausibility of six inferences need to be verified to build a validity argument for a language test, including: *domain definition, evaluation, generalization, explanation, extrapolation*, and *utilization*. Also included in the framework are the key warrants that license each inference and its underlying assumptions. This framework was adopted as the guiding theoretical framework of this review study in the sense that each article collected for this study was classified into one or several of these six inferences in the framework. As such, it is necessary to briefly explain these inferences in Figure 1 in the context of speaking assessment. The explanation of the inferences, together with their warrants and assumptions, is largely based on Chapelle et al. (2008) and Knoch and Chapelle (2018). To facilitate readers' understanding of these inferences, we use the TOEFL speaking test as an example to provide an illustration of the warrants, key assumptions, and backings for each inference.

The first inference, *domain definition*, links the target language use (TLU) domain to test takers' observed performance on a speaking test. The warrant supporting this inference is that observation of test takers' performance on a speaking test reveals the speaking abilities and skills required in the TLU domain. In the case of the TOEFL speaking test, the TLU domain is the English-medium institutions of higher education. Therefore, the plausibility of this inference hinges on whether observation of test takers' performance on the speaking tasks reveals essential academic speaking abilities and skills in English-medium universities. An important assumption underlying this inference is that speaking tasks that are representative of language use in English-medium universities can be identified and simulated. Backings in support of this assumption can be collected through interviews with academic English experts to investigate speaking abilities and skills that are required in English-medium universities.

The warrant for the next inference, *evaluation*, is that test takers' performance on the speaking tasks is evaluated to provide observed scores which are indicative of their academic speaking abilities. The first key assumption underlying this warrant is that the rating scales for the TOEFL speaking test function as intended by the test provider. Backings for this assumption may include: a) using statistical analyses (e.g., many-facets Rasch measurement, or MFRM) to investigate the functioning of the rating scales for the speaking test; and b) using qualitative methods (e.g., raters' verbal protocols) to explore raters' use of the rating scales for the speaking test. Another assumption for this warrant is that raters provide consistent ratings on each task of the speaking test. Backing for this assumption typically entails the use of statistical analyses to examine rater reliability on each task of the speaking test. The third assumption is that detectable rater characteristics do not introduce systematic construct-irrelevant variance into their ratings of test takers' performance. Bias analyses are usually implemented to explore whether certain rater characteristics (e.g., experience, L1 background) interact with test taker characteristics (e.g., L1 background) in significant ways.

The third inference is *generalization*. The warrant that licenses this inference is that test takers' observed scores reflect their expected scores over multiple parallel versions of the speaking test and across different raters. A few key assumptions that underlie this inference include: a) a sufficient number of tasks are included in the TOEFL speaking test to provide stable estimates of test takers' speaking ability; b) multiple parallel versions of the speaking test feature similar levels of difficulty and tap into similar academic English speaking constructs; and c) raters rate test takers' performance consistently at the test level. To support the first assumption, generalizability theory (i.e., G-theory) analyses can be implemented to explore the number of tasks that is required to achieve the desired level of reliability. For the second assumption, backings can be collected through: (a) statistical analyses to ascertain whether multiple parallel versions of the speaking test have comparable difficulty levels; and (b) qualitative methods such as expert review to explore whether the parallel versions of the speaking test tap into similar academic English speaking constructs. Backing of the third assumption typically entails statistical analyses of the scores that raters have awarded to test takers to examine their reliability at the test level.

The fourth inference is *explanation*. The warrant of this inference is that test takers' expected scores can be used to explain the academic English speaking constructs that the test purports to assess. The key assumptions for this inference include: (a) features of the spoken discourse produced by test takers on the TOEFL speaking test can effectively distinguish L2 speakers at different proficiency levels; (b) the rating scales are developed based on academic English speaking constructs that are clearly defined; and (c) raters' cognitive processes when rating test takers' spoken performance are aligned with relevant theoretical models of L2 speaking. Backings of these three assumptions can be collected through: (a) discourse analysis studies aiming to explore the linguistic features of spoken discourse that test takers produce on the speaking tasks; (b) expert review of the rating scales to ascertain whether they reflect relevant theoretical models of L2 speaking proficiency; and (c) rater verbal protocol studies to examine raters' cognitive processes when rating performance on the speaking test.

The fifth inference in the framework is *extrapolation*. The warrant that supports this inference is that the speaking constructs that are assessed in the speaking test account for test takers' spoken performance in English-medium universities. The first key assumption underlying this warrant is that test takers' performance on the TOEFL speaking test is related to their ability to use language in English-medium universities. Backing for this assumption is typically collected through correlation studies, that is, correlating test takers' performance on the speaking test with an external criterion representing their ability to use language in the TLU domains (e.g., teachers' evaluation of students' speaking proficiency of academic English). The second key assumption for *extrapolation* is that raters' use of the rating scales reflects how spoken performance is evaluated in English-medium universities. For this assumption, qualitative studies can be undertaken to compare raters' cognitive processes with those of linguistic laypersons in English-medium universities such as subject teachers.

The last inference is *utilization*. The warrant supporting this inference is that the speaking test scores are communicated in appropriate ways and are useful for making decisions. The assumptions that underlie the warrant include: (a) the meaning of the TOEFL speaking test scores is clearly interpreted by relevant stakeholders, such as admissions officers, test takers, and teachers; (b) cut scores are appropriate for making relevant decisions about students; and (c) the TOEFL speaking test has a positive influence on English teaching and learning. To collect the backings for the first assumption, qualitative studies (e.g., interviews, focus groups) can be conducted to explore stakeholders' perceptions of how the speaking test scores are communicated. For the second assumption, standard setting studies are often implemented to interrogate the appropriateness of cut scores. The last assumption is usually investigated through test washback studies, exploring how the speaking test influences English teaching and learning practices.

The framework was used in the validation of the revised TOEFL, as reported in Chapelle et al. (2008), as well as in low-stakes classroom-based assessment contexts (e.g., Chapelle et al., 2015). According to Chapelle et al. (2010), this framework features several salient advantages over other alternatives. First, given the dynamic and context-mediated nature of language ability, it is extremely challenging to use the definition of a language construct as the basis for building the validity argument. Instead of relying on an explicit definition of the construct, the argument-based approach advocates the specification of a network of inferences, together with their supporting warrants and underlying assumptions that link test takers' observed performances to score interpretation and use. This framework also makes it easier to formulate validation research plans. Since every assumption is associated with a specific inference, research questions targeting each assumption are developed ‘in a more principled way as a piece of an interpretative argument' (Chapelle et al., 2010, p. 8). As such, the relationship between validity argument and validation research becomes more apparent. Another advantage of this approach to test validation it that it enables the structuring and synthesis of research results into a logical and coherent validity argument, not merely an amalgamation of research evidence. By so doing, it depicts the logical progression of how the conclusion from one inference becomes the starting point of the next one, and how each inference is supported by research. Finally, by constructing a validity argument, this approach allows for a critical evaluation of the logical development of the validity argument as well as the research that supports each inference. In addition to the advantages mentioned above for test validation research, this framework is also very comprehensive, making it particularly suitable for this review study.

By incorporating this argument-based validation framework in a narrative review of the published research on speaking assessment, this study aims to address the following research questions:

RQ1. How does the published research on speaking assessment represent the six inferences in the argument-based validation framework?
RQ2. What are the speaking assessment topics that constituted the focus of the published research?
RQ3. What methods did researchers adopt to collect backings for the assumptions involved in each inference?

## Methods

This study followed the research synthesis steps recommended by Cooper et al. (2019), including: (1) problem formation; (2) literature search; (3) data evaluation; (4) data analysis; (5) interpretation of results; and (6) public presentation. This section includes details regarding article search and selection, and methods for synthesizing our collected studies.

### Article Search and Selection

We collected the articles on speaking assessment that were published in *LT* from 1984^1^ to 2018 and *LAQ* from 2004 to 2018. These two journals were targeted because: (a) both are recognized as leading high-impact journals in the field of language assessment; (b) both have an explicit focus on assessment of language abilities and skills. We understand that numerous other journals in the field of applied linguistics or educational evaluation also publish research on speaking and its assessment. Admittedly, if the scope of our review extends to include more journals, the findings might be different; however, given the high impact of these two journals in the field, a review of their published research on speaking assessment in the past three decades or so should provide sufficient indication of the directions in assessing speaking proficiency. This limitation is discussed at the end of this paper.

**Figure 2:**
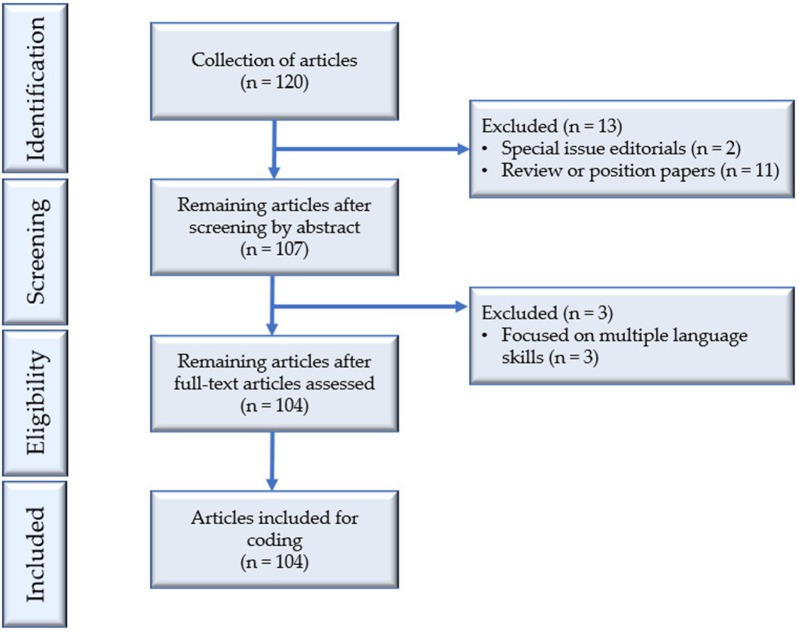
PRISMA flowchart of article search and collection.

The PRISMA flowchart in Figure 2 illustrates the process of article search and selection in this study. A total of 120 articles were initially retrieved through manually surveying each issue in the electronic archives of the two journals, containing all articles published in *LT* from 1984 to 2018 and *LAQ* from 2004 to 2018. Two inclusion criteria were applied: (a) the article had a clear focus on speaking assessment. Articles that targeted the whole language test involving multiple skills were not included; (b) the article reported an empirical study in the sense that it investigated one or more aspects of speaking assessment through the analysis of data from either speaking assessments or designed experimental studies.

Through reading the abstracts carefully, 13 articles were excluded from our analysis, with two special issue editorials and 11 review or position papers. A further examination of the remaining 107 articles revealed that three of them involved multiple language skills, suggesting a lack of primary focus on speaking assessment. These three articles were therefore excluded from our analysis, yielding 104 studies in our collection. Of the 104 articles, 73 (70.19%) were published in *LT* and 31 (29.81%) were published in *LAQ*. All these articles were downloaded in PDF format and imported into NVivo 12 (QSR, 2018) for analysis.

### Data Analysis

To respond to RQ1, we coded the collected articles into the six inferences in the argument-based validation framework based on the focus of investigation for each article, which was determined by a close examination of the abstract and research questions. If the primary focus did not emerge clearly in this process, we read the full text. As the coding progressed, we noticed that some articles had more than one focus, and therefore should be coded into multiple inferences. For instance, Sawaki (2007) interrogated several aspects of an L2 speaking test that were considered as essential to its construct validity, including the interrelationships between the different dimensions of spoken performance and the reliability of test scores. The former was considered as pertinent to the *explanation* inference, as it explores the speaking constructs through the analysis of test scores; the latter, however, was deemed more relevant to the *generalization* inference, as it concerns the consistency of test scores at the whole test level (Knoch and Chapelle, 2018). Therefore, this article was coded into both *explanation* and *generalization* inference.

To answer RQ2, the open coding method (Richards, 1999) was employed to explore the speaking assessment topics that constituted the focus of each article in our collection. This means that a coding scheme was not specified *a prior*; rather, it was generated through examining the abstracts or full texts to determine the topics and subtopics. RQ3 was investigated through coding the research methods that were employed by speaking assessment researchers. A broad coding scheme consisting of three categories was employed to code the research methods: (a) quantitatively oriented; (b) qualitatively oriented; and (c) mixed methods with both quantitative and qualitative orientations. Next, the open coding method was adopted to code the specific methods that were utilized under each broad category. Matrix coding analysis (Miles et al., 2014) was subsequently implemented in NVivo to explore the relationships between the speaking assessment topics, research methods and the six inferences in the argument-based validation framework. This would enable us to sketch the broad patterns of: (a) which topics on speaking assessment tended be investigated under each of the six inferences; (b) which research methods were frequently employed to collect the backings for the assumptions that underlie each inference.

The coding process underwent three iterative stages to ensure the reliability of the coding results. First, both authors coded 10 articles selected randomly from the dataset independently and then compared their coding results. Differences in coding results were resolved through discussion. Next, the first author coded the rest of the articles in NVivo, using the coding scheme that was generated during the first stage while adding new categories as they emerged from the coding process. Finally, the second author coded 20 articles (19.23%) which were randomly selected from the dataset, using the coding scheme that was determined during the second stage. Intercoder agreement was verified through calculating Cohen's kappa statistic in NVivo (*k* = 0.93), which suggested satisfactory coding reliability.

## Results and Discussion

Overall, our coding results indicate that a wide range of research was conducted of speaking assessment to interrogate the six inferences in the argument-based validation framework. These studies cover a variety of research topics, employing quantitative, qualitative, and mixed research methods. In this section, we describe and discuss the analysis results through showcasing the broad patterns that emerged from our coding process. Illustrative studies are used as appropriate to exemplify the research that was undertaken in assessing speaking proficiency.

### Representation of the Published Research in the Six Inferences

Table 1 presents the representation of the published research in the six inferences. As indicated in this table, most of our collected articles were categorized into the three inferences of *evaluation* (*n* = 42, 40.38%), *generalization* (*n* = 42, 40.38%), and *explanation* (*n* = 50, 48.08%); in contrast, a much smaller number of studies targeted the other three inferences of *domain description* (*n* = 4, 3.85%), *extrapolation* (*n* = 7, 6.73%), and *utilization* (*n* = 5, 4.81%). Despite the highly skewed representation of the published research in the six inferences, the findings were not entirely surprising. According to the argument-based validation framework (Chapelle et al., 2008), backings in support of the assumptions that underlie the three inferences of *evaluation, generalization*, and *explanation* relate to almost all key components in the assessment of speaking proficiency, including rater effects, rating scale, task features or administration conditions, interlocutor effects in speaking tasks such as paired oral, interview or group discussion, and features of produced spoken discourse. These components essentially represent the concerns surrounding the development, administration, and validation of speaking assessment (e.g., McNamara, 1996; Fulcher, 2015a). Take the inference of *evaluation* as an example. In the argument-based validation framework, this inference pertains to the link from the observation of test takers' performance on a speaking test to their observed scores. As mentioned previously (see section Theoretical Framework), backings in support of the key assumptions underlying this inference include an evaluation of rating scales as well as rater effects at the task level. Given the pivotal role that raters and rating scales play in speaking assessment (e.g., Eckes, 2011), it is not surprising to observe a reasonably high proportion of studies exploring the plausibility of this inference. Almost half of our collected articles (*n* = 50, 48.08%) interrogated the *explanation* inference. This finding can be interpreted in relation to the centrality of understanding the construct in language test development and validation (e.g., Alderson et al., 1995; Bachman and Palmer, 1996), which lies at the core of the *explanation* inference.

**Table 1:** Representation of the published research in the six inferences (*n* = 104).

**Inferences**	**Number of articles**	
	***n***	**%**
• Domain description	4	3.85
• Evaluation	42	40.38
• Generalization	42	40.38
• Explanation	50	48.08
• Extrapolation	7	6.73
• Utilization	5	4.81

*Thirty-nine articles (37.50%) were coded into multiple inferences, of which 34 (32.69%) were coded into two inferences and five (4.81%) into three inferences*.

One possible explanation for the limited research on *domain description* is related to the journals that formed the basis for this review study. Both *LT* and *LAQ* have an explicit focus on language assessment, whereas in many cases, exploration of language use in TLU domains, which is the focus of *domain description*, might be reported as needs assessment studies in test development reports, which were beyond the purview of this study. Another plausible explanation, as pointed out by one of the reviewers, might lie in the lack of theoretical sophistication regarding this inference. The reason why few studies targeted the *extrapolation* inference might be attributable to the challenges in pinpointing the external criterion measure, or in collecting valid data to represent test takers' ability to use language in TLU domains. These challenges could be exacerbated in the case of speaking ability due to its intangible nature, the various forms that it may assume in practice, and the different conditions under which it happens. Similarly, very few studies focused on the *utilization* inference which concerns the communication and use of test scores. This could relate to the fact that test washback or impact studies have to date rarely focused exclusively on speaking assessment (Yu et al., 2017). Speaking assessment researchers should consider exploring this avenue of research in future studies, particularly against the backdrop of the increasingly extensive application of technology in speaking assessment (Chapelle, 2008).

### Speaking Assessment Topics

Table 2 presents the matrix coding results of speaking assessment topics and the six inferences in the argument-based validation framework. It should be noted that some of the frequency statistics in this table are over-estimated because, as mentioned previously, some articles were coded into multiple inferences; however, this should not affect the general patterns that emerged from the results in a significant way. The topics that emerged from our coding process are largely consistent with the themes that Fulcher (2015a) identified in his review of speaking assessment research. One noteworthy difference is many-facets Rasch measurement (MFRM), a topic in Fulcher (2015a) but was coded as a research method in our study (see section Research Methods). In what follows, we will focus on the three topics which were most frequently investigated by speaking assessment researchers, namely, speaking constructs, rater effects, and factors that affect speaking performance, as examples to illustrate the research that was undertaken of speaking assessment.

**Table 2:** Matrix coding results of inferences and speaking assessment topics (*n* = 104).

**Topics**	**Domain description**		**Evaluation**		**Generalization**		**Explanation**		**Extrapolation**		**Utilization**	
	***n***	**%**	***n***	**%**	***n***	**%**	***n***	**%**	***n***	**%**	***n***	**%**
(1) Speaking constructs (*n* = 47)	0	0.00	8	7.69	11	10.58	39	37.50	5	4.81	0	0.00
(2) Rater effects (*n* = 39)	0	0.00	27	25.96	23	22.12	14	13.46	0	0.00	0	0.00
(3) Factors that affect test performance (*n* = 30)	0	0.00	9	8.65	19	18.27	13	12.50	0	0.00	0	0.00
(4) Speaking test design (*n* = 14)	2	1.92	9	8.65	4	3.85	8	7.69	1	0.96	0	0.00
(5) Test score generalizability (*n* = 7)	0	0.00	3	2.88	7	6.73	2	1.92	0	0.00	0	0.00
(6) Rating scale evaluation (*n* = 6)	2	1.92	4	3.85	2	1.92	2	1.92	0	0.00	0	0.00
(7) Test use (*n* = 5)	0	0.00	0	0.00	0	0.00	0	0.00	1	0.96	5	4.81

*(1) The total number in the left column exceeds 104 because some articles were coded into multiple topic areas; (2) the total numbers of the rows exceed the numbers reported in the left column because some articles in these topic areas were coded into multiple inferences*.

### Speaking Constructs

Table 2 shows that “speaking constructs” (*n* = 47) is the topic that was investigated most frequently in our collected studies. Matrix coding results indicate that this topic area appears most frequently under the inference of *explanation* (*n* = 39, 37.50%). The importance of a clear understanding of the construct cannot be overemphasized in language test development and validation (e.g., Alderson et al., 1995; Bachman and Palmer, 1996). Indeed, construct definition forms the foundation of several highly influential test validation frameworks in the field (e.g., Messick, 1989; Weir, 2005). Our analysis indicates that considerable research has been dedicated to disentangling various speaking constructs. Two topics that feature prominently in this topic area are the analysis of spoken discourse and interactional competence.

A common approach to investigate the speaking constructs is through the analysis of produced spoken discourse (Carter and McCarthy, 2017), usually focusing on linguistic features that can distinguish test takers at different proficiency levels such as complexity, accuracy, and fluency (e.g., Iwashita, 2006; Gan, 2012; Bosker et al., 2013). Research in this area can provide substantial evidence concerning speaking proficiency. Iwashita (2006), for instance, examined the syntactic complexity of the spoken performance of L2 Japanese learners. Results reveal that learner' oral proficiency could be predicted significantly by several complexity indicators, including T-unit length, the number of clauses per T-unit, and the number of independent clauses per T-unit. In another discourse analysis study, Gan (2012) probed the syntactic complexity of test takers' spoken discourse and examined the relationship between syntactic complexity and task type in L2 speaking assessment. Gan's results show that, compared with the group interaction task, test takers' discourses on the individual presentation task featured longer T-units and utterances as well as significantly greater number of T-units, clauses, verb phrases and words. These discourse analysis studies have implications for understanding speaking proficiency as well as its development and maturity among L2 learners.

International competence (IC) is yet another topic which features prominently in this topic area. Despite the recognized need of including IC in speaking assessment (e.g., Kramsch, 1986, McNamara, 1997), how it should be conceptualized remains a contentious issue. Research has shown that this construct consists of multiple dimensions which is susceptible to the influence of a range of personal cognitive and contextual factors (Galaczi and Taylor, 2018). Our review suggests that IC was approached through analyzing test takers' spoken discourse as well as exploring raters' perspectives. Galaczi (2008), for instance, performed elaborate analyses of test takers' spoken discourse on the paired speaking task in the First Certificate in English (FCE) speaking test. The results led the researcher to conclude that test takers' interactions primarily featured three patterns on paired oral assessment tasks: collaborative, parallel and blended interaction (i.e., a mixture of collaborative/parallel or collaborative/asymmetric features). In a more recent study, Lam (2018) analyzed test takers' spoken discourse on a school-based group oral speaking assessment for the Hong Kong Diploma of Secondary Education (HKDSE) English Language Examination. Instead of exploring IC more broadly, as in Galaczi (2008), this study targeted a particular feature of IC, namely, producing responses contingent on previous speakers' contributions. The analyses pointed to three kinds of conversational actions that underpinned a response contingent on previous speaker's contributions: formulating previous speakers' contributions, accounting for (dis)agreement with previous speakers' ideas and extending previous speakers' ideas.

Some other studies explored the construct of IC from raters' perspectives. A typical study was reported by May (2011) who explored the features that were salient to raters on a paired speaking test. The study identified a repertoire of features which were salient to raters, and hence were potentially integral to the IC construct. Such features include, for example, the ability to manage a conversation, ask for opinion or clarification, challenge or disagree with an interactional partner, and demonstrate effective body language, and interactive listening. While suggesting that IC is a highly complex and slippery construct, these studies have significant implications for clarifying the IC construct and promoting its valid operationalization in speaking assessment. The findings are particularly meaningful in the context where interactive tasks are increasingly used in speaking assessment.

### Rater Effects

Raters play a significant role in speaking assessment; their performance is affected by a host of non-linguistic factors, which are often irrelevant to the speaking constructs of interest, hence causing construct-irrelevant variance (Messick, 1989) or contamination (AERA et al., 2014). Not surprisingly, the next topic area that was most frequently explored by speaking assessment researchers is rater effects (*n* = 39). The studies that focused on this topic were mostly classified into the two inferences of *evaluation* (*n* = 27, 25.96%) and *generalization* (n =23, 22.12%). Knoch and Chapelle (2018) applied the argument-based validation framework to the analysis of rater effects and rating processes in language assessment research. They observed that several important aspects of rater effects could be mapped onto *evaluation* and *generalization* inferences. The key assumptions of the *evaluation* inference relate to the raters' consistency at the task level, the bias that raters display against task types or other aspects of the assessment situation, and the impact of raters' characteristics on the ratings that they assign. When it comes to the *generalization* inference, the key assumptions largely concern raters' consistency at the whole test level and the number of raters that is required to achieve the desired level of consistency. Research on rater effects has significant implications for enhancing both the validity and fairness of speaking assessment (e.g., McNamara et al., 2019).

Two topics that feature prominently in the study of rater effects are the impact of raters' characteristics on their rating behaviors and rater cognition, that is, the cognitive processes that raters engage when assigning scores to a spoken performance. Raters' characteristics such as language background, experience and qualifications may have appreciable impact on their ratings. This topic has attracted considerable research attention as it has implications for test fairness and rater training programs. One such study was reported by Kim (2009) who examined and compared the rating behaviors of native and non-native English teachers when assessing students' spoken performance. The results indicate that native-speaker (NS) and non-native-speaker (NNS) teachers on the whole exhibited similar severity levels and internal consistency; however, in comparison with NNS teachers, NS teachers provided more detailed and elaborate comments on students' performance. The findings generally concur with Zhang and Elder (2011) who compared the rating behaviors of NS and NNS teachers in the context of the College English Test - Spoken English Test (CET-SET), a large-scale high-stakes speaking test in China. Instead of focusing on raters' L1 background, Winke et al. (2013) examined whether raters' accent familiarity, defined as their L2 learning experience, constituted a potential source of bias when they rated test takers' spoken performance. In other words, if a rater studies Chinese as his or her L2, is he or she biased toward test takers who have Chinese as their L1? Their findings indicate that the raters with Spanish or Chinese as their L2 were significantly more lenient toward L1 Spanish and Chinese test takers than they were toward those from other L1 backgrounds. However, in both cases, the effect sizes were small, suggesting that such effect had minimal impact in practice. The results are largely consistent with some other studies in our collection (e.g., Yan, 2014; Wei and Llosa, 2015), which explored a similar topic.

Rater cognition or rating processes constitute yet another important topic under the topic area of “rater effects”. Studies along this line are typically implemented through analyzing raters' verbal protocols to explore their cognitive processes when applying the rating criteria or assigning scores to a spoken performance. Research into raters' cognitive processes can generate valuable insights into the validity of the rating scales as well as the speaking constructs that are being assessed in a speaking test. Findings from these studies have important implications for the revision of rating scales, improving rater training programs, and enhancing the validity and usefulness of the speaking test in focus. In a qualitative study, Kim (2015) explored the rating behaviors of three groups of raters with different levels of experience on an L2 speaking test by analyzing their verbal reports of rating processes. The study revealed that the three groups of raters exhibited varying uses of the analytic rating scales, hence suggesting that experience was an important variable affecting their rating behaviors. Furthermore, an analysis of their performance over time revealed that the three groups of raters demonstrated different degrees of improvement in their rating performance. It should be noted that several studies in our collection examined raters' rating processes with a view to either complementing or accounting for the quantitative analyses of speaking test scores. For instance, both Kim (2009) and Zhang and Elder (2011), two studies which were reviewed previously, investigated raters' rating processes, and the findings significantly enriched our understanding of the rating behaviors of raters from different backgrounds.

### Factors That Affect Spoken Performance

The third topic area that emerged from our coding process is “factors that affect spoken performance” (*n* = 30). As shown in Table 3, most of the studies in this topic area were classified into the inference of *generalization* (*n* = 19, 18.27%). This is understandable as factors such as task features, administration conditions, and planning time might affect the generalizability of speaking test scores. Indeed, understanding factors that affect test performance has long since been one of the central concerns for language assessment research as a whole (e.g., Bachman, 1990; Bachman et al., 1995). Research along this line has implications for speaking test development and implementation, and for test score interpretation and use. Our coding analyses indicate that a range of factors have been explored by speaking assessment researchers, of which ‘interlocutor effects' features most prominently. This could be related to the increasingly widespread use of interviews, paired oral or group discussion tasks to assess speaking ability in applied linguistics and language pedagogy. A notable advantage with these assessment formats lies in the unscripted and dynamic nature of the interactions involved, which is key to increasing the authenticity of speaking assessments. Nonetheless, interlocutor characteristics, such as gender, proficiency levels, personality, and styles of interaction might have considerable impact on test takers' spoken performance, thus impinging on the validity, fairness and overall usefulness of these tasks.

**Table 3:** Matrix coding results of research methods and inferences (*n* = 104).

**Methods**	**Domain description**		**Evaluation**		**Generalization**		**Explanation**		**Extrapolation**		**Utilization**	
	***n***	**%**	***n***	**%**	***n***	**%**	***n***	**%**	***n***	**%**	***n***	**%**
QUAN (*n* = 50)	0	0.00	21	20.19	27	25.96	18	17.31	3	2.88	1	0.96
• ANOVA or regression (*n* = 34)	0	0.00	13	12.50	14	13.46	15	14.42	2	1.92	3	2.88
• Rasch (*n* = 28)	0	0.00	19	18.27	20	19.23	9	8.65	0	0.00	0	0.00
• Correlation (*n* = 20)	1	0.96	7	6.73	9	8.65	10	9.62	4	3.85	1	0.96
• G-theory (*n* = 7)	0	0.00	4	3.85	7	6.73	2	1.92	0	0.00	0	0.00
• EFA (*n* = 5)	0	0.00	4	3.85	3	2.88	3	2.88	0	0.00	1	0.96
• SEM (*n* = 5)	0	0.00	2	1.92	3	2.88	2	1.92	1	0.96	0	0.00
• Cluster analysis (*n* = 2)	0	0.00	1	0.96	0	0.00	1	0.96	0	0.00	1	0.96
QUAL (*n* = 23)	3	2.88	4	3.85	3	2.88	16	15.38	2	1.92	0	0.00
• Discourse analysis (*n* = 25)	1	0.96	6	5.78	6	5.78	20	19.23	2	1.92	0	0.00
• Interview/Focus group (*n* = 11)	4	3.85	6	5.78	2	1.92	4	3.85	1	0.96	0	0.00
• Written comments (*n* = 11)	0	0.00	5	4.81	6	5.78	5	4.81	0	0.00	2	1.92
• Verbal protocols (*n* = 10)	1	0.96	7	6.73	2	1.92	5	4.81	0	0.00	0	0.00
• Eye-tracking (*n* = 1)	0	0.00	0	0.00	0	0.00	1	0.96	0	0.00	0	0.00
MIXED (*n* = 31)	1	0.96	17	16.35	12	11.53	16	15.38	2	1.92	4	3.85

*(1) QUAL, Quantitative; QUAL, Qualitative; G-theory, Generalizability theory; EFA, Exploratory factor analysis; SEM, Structural equation modeling; (2) the total number in the left column exceeds 104 because some articles used multiple methods; (3) the total numbers of the rows exceed the numbers reported in the left column because some articles using these methods were coded into multiple inferences*.

An earlier study on interlocutor effects was reported by McNamara and Lumley (1997) who examined the potential impact of interlocutor characteristics on test scores in the context of the Occupational English Test (OET), a high-stakes speaking test for health professionals in Australia. Their study indicated that interlocutor characteristics had some influence on the ratings that test takers received. For example, they found that raters tended to compensate for interlocutors' incompetence in conducting the speaking test; in other words, if an interlocutor was perceived as less competent, test takers tended to receive higher ratings than expected. In addition, they also observe that an interlocutor's ability to build rapport with test takers had a positive effect on the ratings that test takers received. In another study, Brown (2003) probed the effects of interlocutor characteristics on test takers' performance in the context of a conversational interview. She performed elaborate analyses of the interactions between the interviewers (i.e., interlocutors) and test takers, revealing that the interlocutors differed quite significantly in terms of: (a) how they structured topical sequences; (b) their questioning technique; and (c) how they provided feedback and built rapport with test takers. Further analyses uncovered that interviewer styles had quite significant impact on the ratings that test takers received. Resonating with McNamara and Lumley (1997), the findings of this study again call for the reconceptualization of speaking proficiency.

Several other studies focused on the effects of interaction partners in paired or group oral tasks on spoken performance. (Ockey, 2009), for instance, investigated the potential effects of group member's assertiveness levels on spoken performance on a group discussion task. Results confirmed that test takers' assertiveness levels had an impact on the scores that they received. Specifically, assertive test takers were awarded higher scores than expected when grouped with non-assertive test takers; this trend, however, was reversed when they were grouped with test takers with similar assertiveness levels. A plausible explanation could be that raters viewed assertive test takers more positively when other members in the groups were non-assertive, whereas more negatively when other group members, who were also assertive, competed to be the leaders in the interactions. This study reiterates the co-constructed nature of speaking proficiency. Despite the research that has been undertaken of interlocutor effects, controversy remains as to whether this variation is part of the speaking construct and therefore should be incorporated in the design of a speaking test or it should be controlled to such an extent that it poses minimal threat to the reliability and fairness of speaking test scores (Fulcher, 2015a).

In addition to the three topics above, researchers also explored speaking test design (*n* = 14) in terms of the task features (e.g., Wigglesworth and Elder, 2010; Ahmadi and Sadeghi, 2016) and the use of technology in speaking test delivery (e.g., Nakatsuhara et al., 2017; Ockey et al., 2017). The next topic is test score generalizability (*n* = 7), typically investigated through G-theory analysis (e.g., Lee, 2006; Sawaki, 2007; Xi, 2007). Furthermore, six studies in our collection evaluated the rating scales for speaking assessments, including comparing the effectiveness of different types of rating scales (e.g., Hirai and Koizumi, 2013), and examining whether a rating scale functioned as intended by the test developer (e.g., Isaacs and Thomson, 2013). Finally, five studies focused on the use of speaking assessments, mainly relating to test takers' perceptions of speaking assessments (e.g., Scott, 1986; Qian, 2009) and standard setting studies to determine the cut scores for certain purposes (e.g., Pill and McNamara, 2016).

### Research Methods

Table 3 presents the matrix coding results of research methods and inferences. As indicated in this table, quantitative research methods were more frequently employed by speaking assessment researchers (*n* = 50), in comparison with qualitative methods (*n* = 23). It is worth noting that a number of studies (*n* = 31) utilized mixed methods design, which features a combination of both quantitative and qualitative orientations.

Table 3 indicates that quantitative methods were most frequently used to collect backings in support of the *evaluation* (*n* = 21, 20.19%) and *generalization* inferences (*n* = 27, 25.96%). This finding can be interpreted in relation to the key assumptions that underlie these two inferences (see section Theoretical Framework). According to the argument-based validation framework, the assumptions of these two inferences largely concern rater consistency at task and whole-test level, the functioning of the rating scales, as well as the generalizability of speaking test scores across tasks and raters. Understandably, quantitative methods are widely used to collect the backings to test these assumptions. In addition to the overall representation of quantitative methods in speaking assessment research, we also went a step further to examine the use of specific quantitative methods. As shown in Table 3, while traditional data analysis methods such as ANOVA or regression (*n* = 34) continued to be utilized, mainly in the interrogation of the inferences of *evaluation* (*n* = 13, 12.50%), *generalization* (*n* = 14, 13.46%), and *explanation* (*n* = 15, 14.42%), Rasch analysis methods were also embraced by speaking assessment researchers (*n* = 28). Note that Rasch analysis is an overarching term which encompasses a family of related models, among which the many-facets Rasch model (MFRM) is frequently used in speaking assessment (e.g., McNamara and Knoch, 2012). As an extension of the basic Rasch model, the MFRM allows for the inclusion of multiple aspects or facets in a speaking context (e.g., rater severity, task difficulty, difficulty of rating scales). Furthermore, compared with traditional data analysis methods such as correlation and ANOVA which can only provide results at the group level, the MFRM can provide both group- and individual-level statistics (Eckes, 2011). This finding concurs with Fulcher (2015a) who identified the MFRM as an important theme in speaking assessment. It also resonates with the observation of Fan and Knoch (2019, p. 136) who commented that Rasch analysis has indeed become “one of the default methods or analysis techniques to examine the technical quality of performance assessments.” The power of Rasch analysis in speaking assessment research is best illustrated by studies such as Bonk and Ockey (2003), Eckes (2005), and Winke et al. (2013), among others, all of which examined rater effects on speaking assessments in different contexts. Finally, G-theory (*n* = 7) and structural equation modeling (*n* = 5), two complicated quantitative methods, were also utilized by speaking assessment researchers.

In terms of qualitative research methods, discourse analysis is the one which was most frequently employed by speaking assessment researchers (*n* = 25). Matrix coding results indicate that this method features most prominently under the inference of *explanation* (*n* = 20, 19.23%). This finding is aligned with the key assumptions that underlie the *explanation* inference, namely, (a) features of the spoken discourse produced by test takers can effectively distinguish L2 speakers at different proficiency levels, and (b) raters' cognitive processes are consistent with the theoretical models of L2 speaking, both entailing the use of discourse analysis method to explore test takers' spoken responses and raters' rating processes. Importantly, our analysis results indicate that conversation analysis (CA) was the method that appeared frequently under the category of “discourse analysis.” This is best represented by studies such as Galaczi (2008), Lam (2018), and Roever and Kasper (2018), all endeavoring to elucidate the construct of interactional competence. As a data analysis method, CA provides speaking researchers with a principled and intricate approach to analyze the interactions between test takers and examiners in interview, paired oral, or group discussion tasks. Table 3 shows that some other qualitative methods were also quite frequently used by speaking researchers, including interview/focus groups (*n* = 11), written comments (*n* = 11), and verbal protocol reports (*n* = 10). These research methods were typically adopted following the quantitative analyses of test takers' scores, which explains the increasingly widespread use of mixed methods in speaking assessment research (*n* = 31). The finding could find resonance in the observation that mixed method research has been gaining momentum in language assessment research more broadly (e.g., Turner, 2013; Jang et al., 2014; Moeller et al., 2016). As shown in Table 3, mixed-methods design is most frequently employed to collect backings in support of the inferences of *evaluation* (*n* = 17, 16.35%) and *explanation* (*n* = 16, 15.38%). For the *evaluation* inference, mixed method design was often utilized to research rater effects where quantitative and qualitative analyses were used sequentially to examine rating results and processes. When it comes to the *explanation* inference, researchers tended to use a combination of quantitative and qualitative analyses to explore the differences in test takers' speaking scores as well as the spoken discourse that they produced.

## Conclusions and Implications

In this study, we conducted a narrative review of published empirical research on assessing speaking proficiency within the argument-based validation framework (Chapelle et al., 2008). A total of 104 articles on speaking assessment were collected from *LT* (1984–2018) and *LAQ* (2004–2018), two highly influential journals in the field of language assessment. Following the coding of the collected articles, matrix coding analyses were utilized to explore the relationships between the speaking assessment topics, research methods, and the six inferences in the argument-based validation framework.

The analysis results indicate that speaking assessment was investigated from various perspectives, primarily focusing on seven broad topic areas, namely, the constructs of speaking ability, rater effects, factors that affect spoken performance, speaking test design, test score generalizability, rating scale evaluation, and test use. The findings of these studies have significantly enriched our understanding of speaking proficiency and how assessment practice can be made more reliable and valid. In terms of research methods, it was revealed that quantitative research methods were most frequently utilized by speaking assessment researchers, a trend which was particularly pronounced in the inferences of *evaluation* and *generalization*. Though traditional quantitative methods such as ANOVA, regression, and correlation continued to be employed, Rasch analysis played a potent role in researching speaking assessment. In comparison, qualitative methods were least frequently used, mainly for the interrogation of the *explanation* inference. Mixed-methods design, recognized as “an alternative paradigm” (Jang et al., 2014, p. 123), ranked in the middle in terms of frequency, suggesting its increasingly widespread use in speaking assessment research. This is noteworthy when it comes to the *evaluation* and *explanation* inference.

Despite the abundance of research on speaking assessment and the variety of research topics and methods that emerged from our coding process, we feel that there are several areas which have not been explored extensively by language assessment researchers, and therefore warrant more future research endeavors. First, more studies should be conducted to interrogate the three inferences of *domain description, extrapolation*, and *utilization* in the argument-based validation framework. As indicated in our study, only a small fraction of studies have been dedicated to examining these three inferences in comparison with *evaluation, generalization*, and *explanation* (see Table 2). Regarding *domain description*, we feel that more research could be undertaken to understand task- and domain-specific speaking abilities and communicative skills. This would have significant implications for enhancing the authenticity of speaking assessment design, and for constructing valid rating scales for evaluating test takers' spoken performance. The thick description approach advocated by Fulcher et al. (2011) could be attempted to portray a nuanced picture of speaking ability in the TLU domains, especially in the case of Language for Specific Purposes (LSP) speaking assessment. When it comes to the *extrapolation* inference, though practical difficulties in collecting speaking performance data in the TLU domains are significant indeed, new research methods and perspectives, as exemplified by the corpus-based register analysis approach taken by LaFlair and Staples (2017), could be attempted in the future to enable meaningful comparisons between spoken performance on the test and speaking ability in TLU domains. In addition, the judgments of linguistic layperson may also be employed as a viable external criterion (e.g., Sato and McNamara, 2018). The *utilization* inference is yet another area that language assessment researchers might consider exploring in the future. Commenting on the rise of computer-assisted language assessment, Chapelle (2008, p. 127) argued that “test takers have needed to reorient their test preparation practices to help them prepare for new test items.” As such, it is meaningful for language assessment researchers to explore the impact of computer-mediated speaking assessments and automated scoring systems on teaching and learning practices.

Next, though the topic of speaking constructs has attracted considerable research attention from the field, as evidenced by the analysis results of this study, it seems that we are still far from achieving a comprehensive and fine-grained understanding of speaking proficiency. The results of this study suggest that speaking assessment researchers tended to adopt a psycholinguistic approach, aiming to analyze the linguistic features of produced spoken discourse that distinguish test takers at different proficiency levels. However, given the dynamic and context-embedded nature of speaking, there is a pressing need for a sociocultural perspective to better disentangle the speaking constructs. Using pronunciation as an example, Fulcher (2015b) argued convincingly the inadequacy of a psycholinguistic approach in pronunciation assessment research; rather, a sociocultural approach, which aims to demystify rationales, linguistic or cultural, that underlie (dys)fluency, could significantly enrich our understanding of the construct. Such an approach should be attempted more productively in future studies. In addition, as the application of technology is becoming prevalent in speaking assessment practices (Chapelle, 2008), it is essential to explore whether and to what extent technology mediation has altered the speaking constructs and the implications for score interpretation and use.

We also found that several topics were under-represented in the studies that we collected. Important areas that received relatively limited coverage in our dataset include: (a) classroom-based or learning-oriented speaking assessment; (b) diagnostic speaking assessment; and (c) speaking assessment for young language learners (YLLs). The bulk of the research in our collection targeted large-scale high-stakes speaking assessments. This is understandable, perhaps, because results on these assessments are often used to make important decisions which have significant ramifications for stakeholders. In comparison, scanty research attention has been dedicated to speaking assessments in classroom contexts. A recent study reported by May et al. (2018) aimed to develop a learning-oriented assessment tool for interactional competence, so that detailed feedback could be provided about learners' interactional skills in support of their learning. More research of such a nature is needed in the future to reinforce the interfaces between speaking assessment with teaching and learning practices. In the domain of L2 writing research, it has been shown that simply using analytic rating scales does not mean that useful diagnostic feedback can be provided to learners (Knoch, 2009). Arguably, this also holds true for speaking assessment. In view of the value of diagnostic assessment (Lee, 2015) and the call for more integration of learning and assessment (e.g., Alderson, 2005; Turner and Purpura, 2015), more research could be conducted to develop diagnostic speaking assessments so that effective feedback can be provided to promote L2 learners' speaking development. Finally, young language learners (YLLs) have specific needs and characteristics which have implications for how they should be assessed (e.g., McKay, 2006). This is particularly challenging with speaking assessment in terms of task design, implementation and score reporting. This topic, however, has rarely been explored by speaking assessment researchers and therefore warrants more future research.

In terms of research methods, we feel that speaking assessment researchers should consider exploring more the potentials of qualitative methods which are well-suited to investigating an array of research questions related to speaking assessment. Our analysis results indicate that despite the quite frequent use of traditional qualitative methods such as interviews and focus groups, new qualitative methods that are supported by technology (e.g., eye-tracking) have only recently been utilized by speaking assessment researchers. For example, a recent study by Lee and Winke (2018) demonstrated the use of eye-tracking in speaking assessment through examining test-takers' cognitive processes when responding to computer-based speaking assessment tasks. Eye-tracking is advantageous in the sense that as opposed to traditional qualitative methods such as introspective think-aloud protocols, it causes minimal interference of the test taking process. Our final comment concerns the use of mixed-methods design in speaking assessment research. Despite it being applied quite frequently in researching speaking assessment, it appears that only the sequential explanatory design (i.e., the use of qualitative research to explain quantitative findings) was usually employed. Speaking assessment researchers may consider other mixed methods design options (e.g., convergent parallel design or embedded mixed methods design, see Moeller et al., 2016) to investigate more complex research questions in speaking assessment.

We acknowledge a few limitations with this study. As mentioned previously, we targeted only two highly influential journals in the field of language assessment, namely, *LT* and *LAQ* while aware that numerous other journals in applied linguistics or educational evaluation also publish research on speaking and its assessment. As such, caution needs to be exercised when interpreting the relevant research findings that emerged from this study. Future studies could be undertaken to include more journals and other publication types (e.g., research reports, PhD dissertations) to depict a more representative picture of speaking assessment research. In addition, given the sheer volume of published research on speaking assessment available, our research findings can only be presented as indications of possible trends of the wider publishing context, as reflected in the specific articles we explored. Arguably, the findings might be more revealing if we zoomed in on a few key topics in speaking assessment (e.g., rater effects, speaking constructs), analyzed specific studies on these topics in detail, and compared their findings. Finally, it would be worthwhile to explore how the research on some key topics in speaking assessment has been evolving over time. Such analysis could have provided a valuable reference point to speaking assessment researchers and practitioners. Such a developmental trend perspective, however, was not incorporated in our analysis and could be attempted in future research.

## Data Availability Statement

The datasets generated for this study are available on request to the corresponding author.

## Author Contributions

JF designed the study, collected and coded the data, and drafted the article. XY collected and coded the data, and drafted this article together with JF.

### Conflict of Interest

The authors declare that the research was conducted in the absence of any commercial or financial relationships that could be construed as a potential conflict of interest. The handling editor declared a past collaboration with one of the authors JF.

## Footnotes

^1^*LT* and *LAQ* made their debut in 1984 and 2004, respectively.
